# Perceived factors influencing patient acceptance of reduced surveillance of low-risk pancreatic cysts – A Dutch focus group study

**DOI:** 10.1016/j.pmedr.2025.103272

**Published:** 2025-10-11

**Authors:** Marloes L.J.A. Sprij, Jihane Meziani, Marco J. Bruno, Inge M.C.M. de Kok, Ida J. Korfage, Djuna L. Cahen, on behalf of the PACYFIC-registry work group

**Affiliations:** a Department of Gastroenterology and Hepatology, Erasmus MC, University Medical Center Rotterdam, Rotterdam, the Netherlands; b Department of Public Health, Erasmus MC, University Medical Center Rotterdam, Rotterdam, the Netherlands

**Keywords:** Focus group, Barriers and facilitators, Health belief model, Pancreatic cyst surveillance, Low-risk pancreatic cyst, Pancreatic cancer

## Abstract

**Objective:**

This study aimed to explore how patients perceive less intensive surveillance recommendations for low-risk pancreatic cysts.

**Methods:**

Three semi-structured focus groups with individuals undergoing surveillance for low-risk pancreatic cysts were conducted. Results were thematically analyzed and structured according to the health-belief model, which addresses adoption of health behaviors.

**Results:**

Sixteen Dutch individuals participated, including eight females. The median age was 68 years (range 42–77).

Several knowledge gaps emerged as modifiable barriers to acceptance of reduced surveillance, including limited understanding of pancreatic cancer (PaC) risks, cyst prevalence, cyst-related symptoms, and PaC treatment. Many expressed concerns regarding losing reassurance, with some considering self-organized follow-ups. PaC among family or social circles considerably contributed to reluctance in accepting limited surveillance. Conversely, evidence supporting the safety of less surveillance appeared a strong facilitator. Participants were more willing when their cyst remained stable, because they perceived themselves as low risk. Trust in the medical profession and adequate information provision supported acceptance.

**Conclusion:**

Loss of reassurance was a key barrier to accepting reduced surveillance. Addressing knowledge gaps and providing clear evidence of its safety considerably improved acceptance. These insights can help physicians counsel patients more effectively to improve patient acceptance and facilitate implementation of less rigorous guidelines.

## Highlights

• Loss of potential reassurance was a key barrier to reduced cyst surveillance.
• Knowledge gaps about cysts and pancreatic cancer appeared modifiable barriers.
• Clear evidence supporting safety of less surveillance appeared a strong facilitator.
• Experience with pancreatic cancer heighted resistance to reduced surveillance.

## 1 Background

Pancreatic mucinous cysts have the potential to develop into pancreatic cancer (PaC), which is why guidelines advise lifelong (semi-)annual surveillance with imaging modalities (such as Magnetic Resonance Imaging [MRI] or endoscopic ultrasound) (*Gut*, 2018; Ohtsuka et al., 2024). However, over the past decade, there has been a paradigm shift in pancreatic cyst management, driven by new scientific insights and growing concerns about overdiagnosis and overtreatment. Since 2006, the number of cysts being resected has decreased, while surveillance has increased (*Gut*, 2018; Ohtsuka et al., 2024; Tanaka et al., 2006). The latest international guideline took this a step further by recommending less rigorous surveillance for low-risk cysts (Ohtsuka et al., 2024), recognizing that the risk associated with these so-called ‘trivial’ cysts is minimal. This paves the way for further reduction or even cessation of surveillance for this low-risk group (Chhoda et al., 2023; Han et al., 2024; Meziani et al., 2025). However, the question is how patients, who may have been undergoing surveillance for years and may perceive their pancreatic cysts as a health threat, will respond to such a change in regimen.

To date, no studies have assessed how patients with trivial cysts perceive less intensive surveillance strategies. Given that these patients have been (repeatedly) informed of cysts' malignant potential, reluctance towards less rigorous surveillance may be expected. Also, in general, participants in a surveillance program often prefer more rigorous monitoring, especially when they have become accustomed to it (Ghanouni et al., 2020; Piper et al., 2018; Rainey et al., 2020). Finally, they may be unaware of the risks of overdiagnosis and overtreatment, along with their associated drawbacks.

Understanding the barriers and facilitators to acceptance of reduced surveillance is crucial, as it allows clinicians to address patients' information needs, alleviate concerns, and potentially improve adherence to adapted surveillance policies. Thus, this study aimed to explore patients' attitudes and perceptions towards less intensive pancreatic cyst surveillance strategies.

## 2 Methods

### 2.1 Study design

The current study is part of the PACYFIC registry (Pancreatic Cyst Follow-up, an International Collaboration, www.pacyfic.net). From 2015, this multicenter prospective observational cohort study evaluates the yield of pancreatic cyst surveillance. It includes individuals with neoplastic and unspecified pancreatic cysts – either newly or previously diagnosed or operated upon – who require surveillance as determined by their treating physician. Secondary outcomes include patients' attitudes towards surveillance. The study has been approved by the institutional review board of the Erasmus Medical Center (MEC-2014-021) and is executed following the Declaration of Helsinki.

### 2.2 Focus group setting and participant selection

Three face to face focus group sessions were conducted in June and July 2023 at Erasmus Medical Center (a university hospital) and Amstelland hospital (a regional hospital), both participating centers in the PACYFIC-study. For each focus group session, four to six individuals were selected from the PACYFIC participants. We specifically targeted the lowest risk group, as they will most likely be affected by guideline adaptations. Group members had to be >18 years old, Dutch-speaking, and monitored for low-risk cysts (≤ 1.5 cm in size, without worrisome features and/or high risk stigmata). To ensure a range of views, each focus group session included individuals varying in age, gender, medical and family history, socio-economic status and years under surveillance. Participants were invited by a study team member (M.L.J.A. Sprij or D.L. Cahen) via email, face-to-face contact, or telephone, and were provided with further information regarding the study's purpose and background. All participants gave informed consent before taking part in the focus groups.

### 2.3 Data collection

A semi-structured interview guide **(Appendix 1)** was used to explore the experiences and perspectives of the participants. Each focus group session lasted approximately 90 min. Data saturation occurred after the third focus group session, meaning no new themes were identified. The first session was led by a female expert in qualitative research and experienced moderator (I.J. Korfage), accompanied by a female gastroenterologist, with expertise in pancreatic cyst surveillance (Cahen). The second session was moderated by Cahen and supervised by Korfage, while the third session was conducted independently by Cahen. In all sessions, Sprij acted as an observer and took field notes. Participants received details about the moderators' academic backgrounds and research interests. The Consolidated Criteria for Reporting Qualitative Studies: 32-item checklist were applied **(Appendix 2) (****Tong et al., 2007****)**.

### 2.4 Data analysis and health belief model (HBM)

The focus groups were audio recorded and transcribed with personal identifiers removed. After each session, preliminary results were drafted and sent to the participants for member checking. This process allowed them to review the findings and ensure that their perspectives were accurately represented. The transcripts were then independently analyzed by two researchers (Sprij and J. Meziani) using Braun and Clarke's method of thematic analysis (Braun and Clarke, 2006). This method involves the following steps; familiarization with the data, code generating, identification, reviewing, defining and naming themes, and the development of a report presenting the final analysis.

The focus group results were analyzed and structured based on the principles of the Health Belief Model (HBM). This model is a psychological framework designed to explain why individuals adopt specific health behaviors. It focuses on individuals' beliefs about health and illness, as well as the personal and situational factors that shape these beliefs (Rosenstock et al., 1988; Janz and Becker, 1984). In our case, acceptance of reduced surveillance was viewed as a form of health behavior. Self-efficacy was interpreted as participants' confidence in their ability to accept less surveillance. Also, the subthemes were ordered from most to least important based on participants' identification of the most relevant factors. Remaining subthemes were ranked according to frequency and the researchers' assessment of their significance.

The identified themes and final results were discussed with Korfage and Cahen until consensus about the interpretation of study findings was reached. The transcripts were analyzed using ATLAS.ti (version 24.1.1), and demographic data were evaluated using Microsoft Excel (version 2402).

## 3 Results

Together, 16 individuals participated in one of the three focus groups and Table 1 shows their characteristics. Nine invited individuals refused participation, mostly because of work commitments (*n* = 6). Other reasons included unrelated serious health problems (*n* = 1), caregiving responsibilities (n = 1), and concerns about insufficient language proficiency (n = 1).

**Table 1:** Characteristics of the Dutch focus group participants with low-risk pancreatic cysts (2023).

Characteristics	Participants, *n* = 16
Age, years, median (range)	68 (42–77)
Female	8
Caucasian	16
Medical history of cancer	5
Family history of pancreatic cancer	5
Duration of surveillance, years, median (range)	4.1 (0.1–14.1)
Low socioeconomic status	6
Working ≥24 h per week	6

**Fig. 1:**
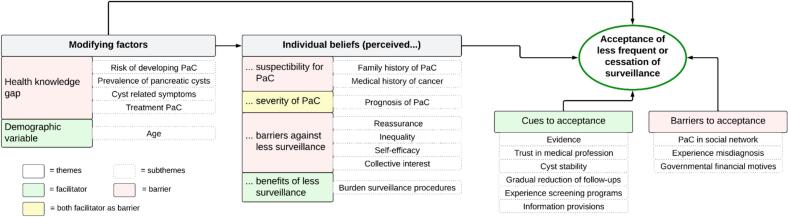
Acceptance of reducing or stopping surveillance of pancreatic cysts, framework based on the Health Belief Model. PaC, pancreatic cancer.

**Fig. 2:**
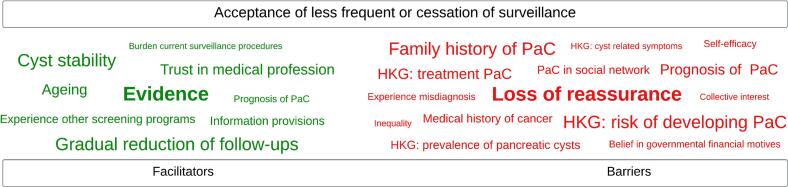
Schematic word cloud depicting the subthemes derived from Dutch focus group participants with low-risk pancreatic cysts (2023), ranked from most to least important and stratified into facilitators and barriers. Ranking is based on participants' identification of most relevant subthemes. Remaining subthemes are ordered according to their frequency of mention and the researchers' assessment of their significance. HKG, health knowledge gap. PaC, pancreatic cancer.

Fig. 1 presents the results structured by the HBM framework and Fig. 2 shows a schematic presentation of the most to least important subthemes affecting acceptance.

### 3.1 Modifying factors

#### 3.1.1 Health knowledge gaps

Participants were unaware that 46 % of the general population has pancreatic cysts, although most sub-centimetre (Kromrey et al., 2018). After addressing this health knowledge gap (HKG) during the focus group sessions, their perspective on surveillance often shifted (Table 2, HKG; pancreatic cyst prevalence).

**Table 2:** Focus group quotations of the Dutch participants with low-risk pancreatic cysts (2023) concerning ‘Modifying factors’.

Theme; subtheme	Quotation
HKG; pancreatic cyst prevalence	*“If half the people in the Netherlands are walking around with small cysts without knowing it, then why should I be monitored.”?*
HKG; risk of developing PaC	*Quotation 1: “If you don't know the percentage is even below 1 %. Well, then you know nothing, so you choose safety.”* *Quotation 2: “If I hear the doctor say now, the percentage of cysts that actually turn into cancer is so minimal, then I think, once every two years is fine.”*
HKG; cyst related symptoms	*Quotation 1: “The thoughts in your head are actually worse than the cyst itself.”* *Quotation 2: “Unless I start having symptoms again, otherwise I'm fine with every two years.”*
HKG; treatment PaC	*“But then maybe you should stop surveilling me. To put it bluntly, even though I'm 53 years old, I'm not fit. I get around with a mobility scooter and can walk a maximum of 500 m per day.”*
Demographical variable; age	*“I'm almost 73, so not extremely old, but if you're, for example, 43, you still have 30 more years to go. As you get older, your life naturally gets shorter. So you might think more easily; oh well, that's a shame.”*

Abbreviations: HKG, Health-knowledge gap; PaC, pancreatic cancer.

The majority of the participants were aware that the risk of developing PaC from a pancreatic cyst is low, but were unable to indicate how low (Table 2, HKG; risk of developing PaC, quotation 1–2). The participants expected that this knowledge gap leads to a preference for more frequent surveillance. After they were informed that the risk is minimal, they became more receptive to reduced surveillance.

Some participants associated their cysts with symptoms and conditions, such as dizziness, kidney dysfunction, and hypoglycemia, which are unrelated to PaC or pancreatic cysts. While they recognized the unlikely connection between the cyst and the symptoms, they remained uncertain about whether the cyst might be responsible (Table 2, HKG; cyst related symptoms, quotation 1). For some, absence of symptoms was key to accepting reduced surveillance (Table 2, HKG; cyst related symptoms, quotation 2).

Most participants were unaware that PaC treatment is strenuous, that it requires good physical health, and that postoperative morbidity is high. After learning about this during the focus groups, the majority agreed that surveillance should be discontinued if a patient's condition is insufficient. Moreover, most participants viewed poor physical fitness as a more important reason to stop surveillance than older age. One participant even reconsidered her suitability for surveillance on the spot, after reflecting on her own fitness level (Table 2, HKG; treatment PaC).

#### 3.1.2 Demographical characteristics

Many participants indicated that, with age, they found discontinued or reduced surveillance more acceptable (Table 2, Demographical variable; age).

### 3.2 Individual beliefs

#### 3.2.1 Perceived susceptibility for PaC

Individuals with a family history of PaC perceived themselves to be at higher risk of developing PaC (Table 3, Perceived susceptibility for PaC; family history of PaC). In addition, those with a history of cancer were particularly concerned about the malignant potential of their cyst (Table 3, Perceived susceptibility for PaC; medical history of cancer). This heightened fear made these individuals less open to reduced surveillance.

**Table 3:** Focus group quotations of the Dutch participants with low-risk pancreatic cysts (2023) concerning ‘Individual beliefs’.

Theme; subtheme	Quotation
Perceived susceptibility for PaC; family history of PaC	*“My father died of PaC at 61. My grandmother, his mother, died of PaC too. So, you always have this fear, maybe I'll be next in line.”*
Perceived susceptibility for PaC; medical history of cancer	*“I've just become more aware, because the melanoma was a close call.”*
Perceived severity of PaC; prognosis of PaC	*“You only notice PaC when it's too late, and if you haven't had check-ups for a while, then you probably already have metastases elsewhere.”*
Barriers against less surveillance; reassurance	*Quotation 1: “We're good for another year.”* *Quotation 2: “I wouldn't have an issue with less frequent check-ups when looking at the data, but emotionally speaking, I would really prefer not to [having less frequent check-ups].”* *Quotation 3: “For now, I find it [annual surveillance] reassuring and safe. Even though I know that if you detect it [PaC], the options for intervention are limited. But yes, it still gives a feeling of safety.”*
Barriers against less surveillance; self-efficacy	*“And if annual surveillance is no longer possible for whatever reason, I'll make sure it happens annually myself, even if it's not proven to help. It gives me a sense of having a bit more control.”*
Barriers against less surveillance; inequality	*“I would indeed have it [annual surveillance] checked in private healthcare, and that doesn't feel fair, because I might have the resources to do that. And then you create inequality.”*
Benefits of less surveillance; burden surveillance procedures	*Quotation 1: “Then you're dealing with it [waiting for MRI results] for three weeks. And, it's strange to say, but I just have diarrhea until I get the results.”* *Quotation 2: “Well, you have to make time for it, but that's really the only thing. I don't think that's a disadvantage, actually. It doesn't outweigh the benefits.”* *Quotation 3: “It costs the hospital a lot too. So then I think differently about it [surveillance frequency], maybe once every three years instead.”*

Abbreviations: PaC, pancreatic cancer.

#### 3.2.2 Perceived severity of PaC

Some participants preferred annual surveillance, fearing that reducing or stopping it might result in PaC being detected too late, which would decrease their chances of survival (Table 3, Perceived severity of PaC; prognosis of PaC). On the other hand, some questioned the effectiveness of surveillance, given the poor prognosis even if PaC is detected early. This contributed to their acceptance of ending surveillance.

#### 3.2.3 Barriers against less surveillance

The majority of participants appreciated the reassurance provided by annual surveillance, as it offered them peace of mind (Table 3, Barriers against less surveillance; reassurance, quotation 1–3). This sense of security made it difficult for them to reduce or stop surveillance, despite of its unproven effectiveness. Some participants were aware that annual surveillance offers only limited protection and they understood that it does not guarantee detecting PaC at a treatable stage. Regardless, they found surveillance reassuring and were reluctant to reduce it.

A few participants also expressed a lack of self-efficacy, indicating that they would pursue annual examinations, regardless of the available evidence (Table 3, Barriers against less surveillance; self-efficacy). One participant raised concerns about inequity in this scenario. She feared that with biennial surveillance, only wealthier individuals will continue annual surveillance through private healthcare, leading to disparities (Table 3, Barriers against less surveillance; inequality).

Some expressed that their involvement in surveillance was driven by a desire to contribute to scientific research and help future patients. In their view, reducing or discontinuing surveillance should not come at the expense of advancing medical knowledge.

#### 3.2.4 Benefits of less surveillance

Some participants found surveillance physically and emotionally burdensome (Table 3, Benefits of less surveillance; burden surveillance procedures, quotation 1). For instance, MRI scans caused discomfort due to claustrophobia or asthma-related breathing difficulties. The waiting period for MRI results was another source of distress. Logistical challenges, such as long travel times for brief consultations and the need to take time off work for appointments, were also highlighted. Despite this, participants generally emphasized that the benefits of surveillance outweigh the disadvantages (Table 3, Benefits of less surveillance; burden surveillance procedures, quotation 2). Some participants also acknowledged that the financial burden on hospitals and insurers made them more open to the idea of reducing surveillance frequency (Table 3, Benefits of less surveillance; burden surveillance procedures, quotation 3).

### 3.3 Cues and barriers to acceptance

#### 3.3.1 Cues to acceptance

For all participants, evidence showing that the risks of reducing or stopping surveillance are minimal was essential to their acceptance. Some explicitly stated the need for precise risk percentages, as scientific evidence gave them confidence (Table 4, Cues to acceptance; evidence). Cyst stability over time was frequently cited as reassuring and made participants more receptive to less frequent surveillance. Moreover, some participants valued cyst stability more highly than a specific risk percentage (e.g., 5 %, 1 %, or 0.1 %) to cease surveillance (Table 4, Cues to acceptance; cyst stability).

**Table 4:** Focus group quotations of the Dutch participants with low-risk pancreatic cysts (2023) concerning ‘Cues and barriers to acceptance’.

Theme; subtheme	Quotation
Cues to acceptance; evidence	*“I want to know a risk percentage because then research has been done, right? Scientific research. And that is the most important thing for me.”*
Cues to acceptance; cyst stability	*“At this moment, I wouldn't want that because I don't know when the cyst appeared. But yes, if it stays stable, then fine, because the risk is very small.”*
Cues to acceptance; gradual reduction of follow-ups	*“If you've been following me every year, and you suddenly say, well, it's no longer necessary, then I think, oh no, is this going to go well? But if you say, now you can come every two years, then three years, then four, then maybe you feel more confident.”*
Cues to acceptance; trust in the medical profession	*“He [the physician] studied for it, I didn't!”*
Cues to acceptance; information provision	*“That in all these years you've never had the same doctor, I find that a very big problem. …And you barely talk to them – just 5* min *of ‘Everything's fine, see you next year’. And now I hear that we're actually supposed to have a proper conversation.”*
Barriers to acceptance; PaC in social network	*“I'd never worried about it [pancreatic cyst] until last year, when a friend passed away from PaC within six weeks. Then I thought, oops. It's good that this [annual surveillance] takes place.”*
Barriers to acceptance; experience misdiagnosis	*“But everyone says the doctor knows best. But the doctor could also be wrong, and I've seen that with my grandmother.”*
Barriers to acceptance; governmental financial motives	*“And I think that's where you really hit a raw nerve with patients. Because you're not a statistic, and you're not a cost calculation.”*

Abbreviations: PaC, pancreatic cancer.

Many participants were generally accustomed to annual follow-ups and found the idea of abruptly stopping surveillance uncomfortable. They preferred a gradual reduction in frequency, as it fostered more trust (Table 4, Cues to acceptance; gradual reduction of follow-ups). Those with experience in other screening or surveillance programs found it more acceptable to reduce or discontinue surveillance, as this is common practice in other programs too.

Trust in the medical profession was another important factor influencing acceptance. Most participants believed that decisions about discontinuing surveillance should be made by healthcare professionals. Some expressed complete trust in such decisions (Table 4, Cues to acceptance; trust in the medical profession).

Participants felt that physicians should start the conversation on reducing surveillance. Preferably, this should be done by a physician with whom a trusted physician-patient-relationship has been established and who takes the time to explain the rationale behind the decision. The absence of a trusted physician, on the other hand, led to brief, superficial conversations lacking depth (Table 4, Cues to acceptance; information provision). Participants showed varied preferences for informational resources, such as risk category visualizations, videos, or brochures. There was no consensus on a preferred format, and preferences appeared to depend on individual needs.

#### 3.3.2 Barriers to acceptance

Participants who had witnessed someone they knew being diagnosed with PaC were more fearful of developing the disease themselves (Table 4, Barriers to acceptance; PaC in social network). They were more aware of the risks and consequences of the condition, making them less willing to stop or reduce surveillance.

Another participant, whose grandmother died of a malignant cyst that had been misdiagnosed, expressed skepticism about the low risk assessment of small, stable cysts. Due to her experience she was not open to reduced surveillance (Table 4, Barriers to acceptance; experience misdiagnosis).

Some participants suspected that cost-saving is the motivation behind less rigorous surveillance strategies. This perception triggered negative emotions, making the idea of reducing or discontinuing surveillance unacceptable to them (Table 4, Barriers to acceptance; governmental financial motives).

## 4 Discussion

To our knowledge, this is the first study to explore patient perspectives influencing acceptance of less rigorous surveillance among individuals with low-risk pancreatic cysts. As this group is likely to face a transition to reduced surveillance, we used a qualitative approach to identify key barriers and facilitators. Consistent with prior studies on risk-based screening (Lippey et al., 2019; He et al., 2018), more barriers than facilitators were identified. Loss of reassurance and a health knowledge gap emerged as prominent barriers. Conversely, closing this knowledge gap and presenting evidence on the safety of reduced surveillance seem to be effective facilitators.

Literature shows that enhancing health knowledge is associated with behavior that is considered healthy by health authorities and physicians, whereas a lack of knowledge hinders it (Sousa et al., 2023; Dickson-Swift et al., 2024). For example, increasing awareness of the harms and benefits of breast cancer screening through decision aids in individuals has been shown to reduce the intention to continue screening beyond the recommend age (Dickson-Swift et al., 2024). The knowledge gaps identified in our study can be addressed by physicians or incorporated in patient information materials. Similar to findings from other studies (Rainey et al., 2019; Bas et al., 2023), providing convincing evidence on the safety of reduced or discontinued surveillance is essential. Additionally, emphasizing cyst stability is crucial, as individuals find this highly reassuring. Notably, false positives resulting in unnecessary major surgery, were not mentioned by the focus group participants, suggesting they were unaware of this potential harm. As physicians inform patients at the start of surveillance, this knowledge may be forgotten over time (Kessels, 2003). Additionally, scientific knowledge continuously evolves, particularly on surveillance effectiveness. Thus, regularly repeating and updating information will improve health literacy.

While participants appeared more open to the idea of discontinuation with increasing age, the majority preferred basing this decision on health status rather than age. Our findings suggest that patients and experts view the benefits of PaC surveillance for fit elderly differently. Experts often argue that surveillance beyond a certain age – particularly in low-risk populations – is not efficient or cost-effective (Ohtsuka et al., 2024). In contrast, patients may believe that preventive measures remain beneficial as long as they are in good health, regardless of age. Proactively discussing physical fitness during consultations might be an effective strategy to prevent over-surveillance for those with declining health. For physically fit elderly, shifting the focus away from strict age criteria and instead using statements such as “*the MRI is not going to help you live longer*” might improve understanding and acceptance (Schoenborn et al., 2017).

For some individuals, reassuring them is more challenging than others. As expected, those with a family history of PaC exhibited strong reluctance towards less intensive monitoring, but hesitation was also noticed among those who had encountered someone with PaC in their social circle. Merely informing these individuals about the safety of reduced surveillance is unlikely to suffice, and additional counseling to address their worries is likely necessary. However, full acceptance may potentially never be achieved despite physicians' efforts.

A notable difficulty in pancreatic cyst surveillance is the inability to actively reduce the associated risk. Although the risk is minimal for low-risk cysts (Chhoda et al., 2023), it remains present and cannot be modified through interventions which may reduce patient anxiety. In contrast, for example, colorectal cancer screening through colonoscopy allows physicians to actively reduce risk by removing polyps, thereby offering a clear risk reduction. While one might argue that individuals should simply comply with reduced surveillance, our findings suggest that this approach may not be well received. Consistent with other studies (Lifford et al., 2013; Jones-Webster et al., 2024), many individuals found it challenging to accept less surveillance because they valued the reassurance provided by favorable results. Some individuals with a lack of self-efficacy, even opted to self-organize private MRIs when a reduction is proposed, a trend also seen in breast cancer screening (Rainey et al., 2020; Rainey et al., 2022). A novel finding was that participants suggested a stepwise reduction in follow-up frequency. Although this approach may enhance acceptance and prevent individuals from seeking private healthcare, it would lead to complex follow-up schedules and unnecessary costs.

When introducing a less intensive surveillance approach, physicians must reframe their original message about the cyst's malignancy risk and need for surveillance. As expected, trust in the medical profession—particularly in a patient's own treating physician— emerges as facilitator. Research highlights the influence of the physician-patient interaction (Peterson et al., 2016; Young et al., 2017). While previous research suggested that discussing discontinuation can damage the patient-physician relationship (Torke et al., 2013), our study found consensus among participants that physicians should start such discussions. Evidence on the effectiveness of specific communication techniques is limited. Patient-centered communication, which prioritizes patient preferences, needs, and values, alongside scientific evidence, offers a promising approach. Commonly used in shared- and informed decision-making (Hashim, 2017; McCormack et al., 2011), this method improves treatment adherence and trust in the physician (Jiang et al., 2024; Schoenthaler et al., 2017). The ask-tell-ask technique is an example of this method, involving open-ended questions to assess a patients' knowledge and concerns, delivering concise and manageable information, and checking for understanding. When individuals express hesitation or resistance, underlying barriers are explored.

Although individuals seem to respond positively to being informed about their risk status (Rainey et al., 2020; Digby et al., 2020), further research is needed to determine the optimal level of detail. The extent of desired information varied among our study participants; ranging from simple assurance of safety to detailed risk assessments. Obviously, overly detailed risk information can be difficult to comprehend. As physicians initiate the conversation regarding less rigorous surveillance and provide the first information about an individual's risk status, we recommend making the informational materials more detailed. This would offer those seeking additional risk details a deeper understanding while potentially saving physicians time during consultations. Preferences for how information is presented also varied, underscoring the need for multiple formats of information delivery (Toes-Zoutendijk et al., 2023).

### 4.1 Strengths and limitations

The primary strength of this study lies in the use of an focus group design rather than individual interviews, which enabled comprehensive discussions that participants might not have considered independently. The deliberate sampling of participants with diverse characteristics, except for ethnicity, across two different hospitals provided valuable insights. Although we used data saturation as a stopping criteria, a recognized and commonly used approach in qualitative research, there remains a possibility that non-response bias have occurred. The majority of the individuals who declined participation were employed, whereas most of the participants were either retired or unemployed. This is a common challenge in qualitative research and may affect the generalizability of our findings.

### 4.2 Clinical implications

Our findings can facilitate smoother implementation of less rigorous pancreatic cyst surveillance. They may assist physicians in tailoring their counseling to better address patient needs and alleviate potential concerns. Also, these insights are valuable for the development patient information materials. However, further research is needed to verify our findings in larger and more diverse populations.

## 5 Conclusion

Focus group discussions identified more barriers than facilitators influencing the acceptance of less frequent pancreatic cyst surveillance among individuals with a low-risk pancreatic cyst. Participants initially expressed resistance, citing the loss of reassurance after favorable results as a primary concern. However, addressing health knowledge gaps and highlighting evidence supporting the safety of reduced surveillance improved acceptance. Addressing the personal experiences or individual beliefs that serve as barriers is crucial, as full acceptance will otherwise be difficult to achieve.

## Author contribution

**Marloes Sprij:** Conceptualization, Methodology, Data collection, Formal analysis, Project administration, Writing – original draft, Writing – review and editing. **Jihane Meziani:** Methodology, Formal analysis, Writing – review and editing. **Inge de Kok**: Conceptualization, Writing – review and editing, Funding acquisition. **Marco Bruno:** Conceptualization, Writing – review and editing, Funding acquisition. **Djuna Cahen:** Conceptualization, Methodology, Data collection, Formal analysis, Writing – review and editing, Supervision, Funding acquisition. **Ida Korfage:** Conceptualization, Methodology, Formal analysis, Writing – review and editing, Supervision. All authors have read and approved the final manuscript.

## CRediT authorship contribution statement

**Marloes L.J.A. Sprij:** Writing – review & editing, Writing – original draft, Visualization, Software, Project administration, Methodology, Investigation, Formal analysis, Data curation, Conceptualization. **Jihane Meziani:** Writing – review & editing, Methodology, Formal analysis. **Marco J. Bruno:** Writing – review & editing, Funding acquisition. **Inge M.C.M. de Kok:** Writing – review & editing, Funding acquisition. **Ida J. Korfage:** Writing – review & editing, Supervision, Methodology, Formal analysis, Conceptualization. **Djuna L. Cahen:** Writing – review & editing, Supervision, Funding acquisition, Formal analysis, Data curation, Conceptualization.

## Declaration of competing interest

The authors declare the following financial interests/personal relationships which may be considered as potential competing interests: All authors, except Marco Bruno, declare that they have no known competing financial interests or personal relationships that could have appeared to influence the work reported in this paper. The author, Marco J. Bruno declare the following conflict of interests which may be considered as potential competing interests: Boston Scientific (Consultant, support for industry and investigator-initiated studies), Cook Medical (Consultant, support for industry and investigator-initiated studies), Pentax Medical (Consultant, support for investigator-initiated studies), Mylan (Support for investigator-initiated studies), Ambu (consultant, support for investigator-initiated studies) and ChiRoStim (Support for investigator-initiated studies).

## Data Availability

Data will be made available on request.

## Appendix A Supplementary data

Supplementary material Image 1
