# Supplementary material for: Perceived factors influencing patient acceptance of reduced surveillance of low-risk pancreatic cysts – A Dutch focus group study
Source: Prev Med Rep. 2025 Oct 11;59:103272. doi: 10.1016/j.pmedr.2025.103272 (PMC12550713; doi:10.1016/j.pmedr.2025.103272)
Supplement: Supplementary file 1 — Supplementary material [file mmc1.docx]

### Appendix 1. Semi-structured topic guide

**Introduction**

1: **Welcome and Introduction Round**

- **Introducing the Moderators**
  - Introduce name and profession
  - Define roles
    - Mention that Djuna (Gastroenterologist) may intervene if incorrect information is shared about the cysts.
- **Introduction round participants**

2: **Important Points Before We Begin**

- Audio recording of the conversation + confidentiality of data. The recorded conversation will be transcribed, and personal data will be omitted.
- Agreement that information shared in this room remains confidential.
- Information on travel expense reimbursement: provide claim form.
- Agreement on informal addressing (using first names).
- Questions from participants.

3: **Purpose of the Study and Focus Group Discussion**

- You have been invited because you undergo monitoring for small pancreatic cysts. We would like to discuss this with you. We are curious about your thoughts and experiences since no prior research has been done on this, and we consider it important.
- First, we want to understand how you experience the current monitoring and its impact on your quality of life.
- Second, we are interested in your thoughts on the risks associated with having a cyst.
- Lastly, we want to hear your views on potentially reducing follow-up examinations or even stopping them altogether.
- Our ultimate goal is to develop a questionnaire on these topics for all PACYIFIC participants with small cysts. To ensure we formulate relevant questions and cover all important aspects, we would like to hear about your experiences, thoughts, and opinions.

**Group Discussion**

- The discussion will last approximately 60 minutes. After 30 minutes, we will offer a short break for refreshments.

**Theme 1: Pancreatic Cyst Monitoring**

1. What do you think about the current monitoring of pancreatic cysts?
   - What are your experiences with the monitoring?
   - Do you find the check-ups bothersome or not at all?
     - If yes, why?
   - What do you think about the frequency of follow-up examinations?
2. What are your reasons for participating in pancreatic cyst monitoring?
   - Are there any reasons or considerations for not participating in the check-ups?
3. Do you see any benefits in participating in pancreatic cyst monitoring?
   - If yes, what are they?
4. Do you see any drawbacks to participating in pancreatic cyst monitoring?
   - If yes, what are they?

- We conduct these check-ups because there is a very small chance that a cyst could develop into pancreatic cancer. This leads us to the next topic.

1. Do you ever worry about developing pancreatic cancer?
   - If yes, how does this concern manifest?
   - Does it affect your daily life?
2. Imagine there are 100 people with the same type of cyst as you. How many of them do you think will develop pancreatic cancer in the next five years?

**Theme 2: Quality of Life**

1. What impact do the pancreatic cyst check-ups have on your life?
   - Daily life
   - Quality of life
2. What impact does having a pancreatic cyst have on your life?
   - Daily life
   - Quality of life

**Theme 3: Fewer or No Follow-up Examinations**

We now move on to the last topic—your thoughts on possibly reducing monitoring. Long-term research has shown that the risk of cancer from small cysts is lower than initially believed. This applies to everyone in this room, meaning that in the future, monitoring of small cysts may be required less frequently or not at all.

1. What are your initial thoughts on reducing the frequency of pancreatic cyst monitoring?
   - What are your reasons for agreeing or disagreeing with fewer check-ups?
2. What are your thoughts if monitoring for small pancreatic cysts were to be discontinued altogether?
3. Under what circumstances would you consider reducing or stopping monitoring to be a good idea?
   - What would be necessary to make you feel comfortable with this decision?
4. Under what circumstances would you *not* consider reducing or stopping monitoring to be a good idea?
   - At what level of cancer risk over five years would you find it acceptable to discontinue monitoring?
5. What kind of information would you like to receive if you were advised to reduce or stop monitoring?
   - How would you prefer to receive this information?

**Closing Questions**

Thank you all for sharing your thoughts today. Are there any other issues or topics you find important that we haven’t yet discussed?

- Ask each participant individually.
- Continue until saturation/no new topics arise, then proceed to closing.

**Conclusion**

- Thank participants for their time and input.
- Ask if they agree with a "member check" (verification of findings).

### Appendix 2. COREQ (COnsolidated criteria for REporting Qualitative research) checklist

A checklist of items that should be included in reports of qualitative research. You must report the page number in your manuscript where you consider each of the items listed in this checklist. If you have not included this information, either revise your manuscript accordingly before submitting or note N/A.

| **Topic** | **Item No.** | **Guide Questions/Description** | **Reported on**  **Page No.** |
| --- | --- | --- | --- |
| **Domain 1: Research team**  **and reﬂexivity** | | | |
| *Personal characteristics* | | | |
| Interviewer/facilitator | 1 | Which author/s conducted the interview or focus group? | 2 |
| Credentials | 2 | What were the researcher’s credentials? E.g. PhD, MD | 1 |
| Occupation | 3 | What was their occupation at the time of the study? | 1 |
| Gender | 4 | Was the researcher male or female? | 2 |
| Experience and training | 5 | What experience or training did the researcher have? | 2 |
| *Relationship with*  *participants* | | | |
| Relationship established | 6 | Was a relationship established prior to study commencement? | 2 |
| Participant knowledge of  the interviewer | 7 | What did the participants know about the researcher? e.g. personal  goals, reasons for doing the research |  |
|  |  |  | 2 |
|  |  |  |  |
| Interviewer characteristics | 8 | What characteristics were reported about the inter viewer/facilitator?  e.g. Bias, assumptions, reasons and interests in the research topic |  |
|  |  |  | 2 |
|  |  |  |  |
| **Domain 2: Study design** | | | |
| *Theoretical framework* | | | |
| Methodological orientation and Theory | 9 | What methodological orientation was stated to underpin the study? e.g. grounded theory, discourse analysis, ethnography, phenomenology,  content analysis |  |
|  |  |  | 2-3 |
|  |  |  |  |
| *Participant selection* | | | |
| Sampling | 10 | How were participants selected? e.g. purposive, convenience,  consecutive, snowball |  |
|  |  |  | 1-2 |
|  |  |  |  |
| Method of approach | 11 | How were participants approached? e.g. face-to-face, telephone, mail,  email |  |
|  |  |  | 2 |
|  |  |  |  |
| Sample size | 12 | How many participants were in the study? | 3 |
| Non-participation | 13 | How many people refused to participate or dropped out? Reasons? | 3 |
| *Setting* | | | |
| Setting of data collection | 14 | Where was the data collected? e.g. home, clinic, workplace | 2 |
| Presence of non-  participants | 15 | Was anyone else present besides the participants and researchers? |  |
|  |  |  | 2 |
|  |  |  |  |
| Description of sample | 16 | What are the important characteristics of the sample? e.g. demographic  data, date |  |
|  |  |  | 2-3, table 1 |
|  |  |  |  |
| *Data collection* | | | |
| Interview guide | 17 | Were questions, prompts, guides provided by the authors? Was it pilot  tested? | 2 |
|  |  |  |  |
| Repeat interviews | 18 | Were repeat inter views carried out? If yes, how many? | 2 |
| Audio/visual recording | 19 | Did the research use audio or visual recording to collect the data? | 2 |
| Field notes | 20 | Were ﬁeld notes made during and/or after the interview or focus group? | 2 |
| Duration | 21 | What was the duration of the inter views or focus group? | 2 |
| Data saturation | 22 | Was data saturation discussed? | 2 |
| Transcripts returned | 23 | Were transcripts returned to participants for comment and/or | 2 |

| **Topic** | **Item No.** | **Guide Questions/Description** | **Reported on**  **Page No.** |
| --- | --- | --- | --- |
|  |  | correction? |  |
| **Domain 3: analysis and**  **ﬁndings** | | | |
| *Data analysis* | | | |
| Number of data coders | 24 | How many data coders coded the data? | 2 |
| Description of the coding  tree | 25 | Did authors provide a description of the coding tree? |  |
|  |  |  | N/A |
|  |  |  |  |
| Derivation of themes | 26 | Were themes identiﬁed in advance or derived from the data? | 2 |
| Software | 27 | What software, if applicable, was used to manage the data? | 3 |
| Participant checking | 28 | Did participants provide feedback on the ﬁndings? | 2 |
| *Reporting* | | | |
| Quotations presented | 29 | Were participant quotations presented to illustrate the themes/ﬁndings?  Was each quotation identiﬁed? e.g. participant number |  |
|  |  |  | 3-7 |
|  |  |  |  |
| Data and ﬁndings consistent | 30 | Was there consistency between the data presented and the ﬁndings? | 3-7, figure 1-2 |
| Clarity of major themes | 31 | Were major themes clearly presented in the ﬁndings? | 3-7, figure 1 |
| Clarity of minor themes | 32 | Is there a description of diverse cases or discussion of minor themes? | 3-7, figure 2 |

Developed from: Tong A, Sainsbury P, Craig J. Consolidated criteria for reporting qualitative research (COREQ): a 32-item checklist for interviews and focus groups. *International Journal for Quality in Health Care*. 2007. Volume 19, Number 6: pp. 349 – 357

**Once you have completed this checklist, please save a copy and upload it as part of your submission. DO NOT include this checklist as part of the main manuscript document. It must be uploaded as a separate file.**
